# Dysadherin specific drug conjugates for the treatment of thyroid cancers with aggressive phenotypes

**DOI:** 10.18632/oncotarget.14904

**Published:** 2017-01-30

**Authors:** Samuel Jang, Xiao-Min Yu, Celina Montemayor-Garcia, Kamal Ahmed, Eric Weinlander, Ricardo V. Lloyd, Ajitha Dammalapati, David Marshall, James R. Prudent, Herbert Chen

**Affiliations:** ^1^ Department of Surgery, University of Wisconsin School of Medicine and Public Health, Madison, WI, USA; ^2^ Department of Surgery, University of Alabama Birmingham School of Medicine, Birmingham, AL, USA; ^3^ Department of Pathology and Laboratory Medicine, University of Wisconsin School of Medicine and Public Health, Madison, WI, USA; ^4^ Centrose LLC, Madison, WI, USA

**Keywords:** thyroid cancer, dysadherin, antibody-drug conjugate, aggressive phenotype, treatment

## Abstract

**Background:**

EDC1 is a novel type of antibody-drug conjugate which binds and inhibits the Na,K-ATPase on the surface of cancer cells expressing dysadherin. The purpose of this study was to determine the expression of dysadherin in different types of thyroid carcinoma, and evaluate the therapeutic potential of EDC1 for thyroid carcinomas.

**Methods:**

Thyroid tissues from 158 patients were examined for dysadherin expression and correlation with clinicopathological features. Thyroid cancer cell lines were examined for the expression of dysadherin and effective dose range of EDC1.

**RESULTS:**

One in 53 benign thyroid tissues and 62% of thyroid cancers expressed dysadherin. All anaplastic and a majority of papillary thyroid cancers overexpressed dysadherin, while 25% of follicular thyroid cancers was found to be positive for dysadherin. Dysadherin expression significantly correlated with extrathyroidal extension and lymph node metastases in papillary thyroid cancer. Five of six human thyroid cancer cell lines analyzed expressed high levels of dysadherin. Of those cells lines sensitive to EDC1, half maximal effective concentrations (EC50) were observed to be between 0.125 nM and 1 nM.

**Conclusions:**

EDC1 showed selective inhibition of growth in thyroid cancer cells with moderate to high expression of dysadherin, thus could be a specific and effective treatment.

## INTRODUCTION

Thyroid cancer is the most common endocrine malignancy and the most rapidly increasing cancer in the U.S [1]. While generally thyroid cancers are highly curable with the recommended treatment of thyroidectomy and radioactive iodine ablation [2], anaplastic thyroid cancer (ATC) and about 20% of well-differentiated thyroid cancers present a more aggressive phenotype of distant metastasis or recurrence associated with increased mortality [3–6]. Many of these aggressive thyroid cancers do not concentrate radioactive iodide from the lack of the sodium/iodide symporter (SLC5A5) [7, 8, 9], resulting in decreased survival [8, 10]. Unlike its well-differentiated counterparts, the prognosis for aggressive thyroid cancers is dismal [6, 11] and new treatment modalities are needed in light of the limited options for these patients [12, 13, 14].

Antibody-drug conjugates (ADC) have been discovered as successful options for treating human malignancies. ADC allows the targetted delivery of a highly cytotoxic agents to antigens specifically expressed by cancer cells but not by normal cells. The high clinical efficacy of two ADCs lead to their approvals by the Food and Drug Administration (FDA); brentuximab vedotin targets CD30 expressed in Hodgkin's lymphoma, and ado-trastuzumab emtansine targets the HER2 antigen expressed in some metastatic breast cancer [15–18]. Similar to CD30 and HER2, dysadherin is a cell membrane glycoprotein recentyly discovered on variety of malignant cells, including thyroid [19], colorectal, pancreatic, esophageal, gastric, cervical, testicular, breast, head and neck carcinomas, and malignant melanoma [20–30]. Although the biological role of dysadherin is still being elucidated, previous studies show that it promotes invasiveness by up regulating CCL2 and down regulating E-cadherin1, enhances the activity of the NF-κB pathway, and promotes metastasis through a mechanism that involves enhanced AKT activation [31, 32].

Interestingly, dysadherin surface expression is limited on normal cells and rarely expressed on non-neoplastic cells of the types associated with dysadherin positive malignancies studied to date. This feature of specificity makes dysadherin a very promising target for ADC development. We therefore utilized a highly potent type of ADC called EDC1. EDC1 is a non-internalizing extracellular drug conjugate composed of an anti-dysadherin antibody (NCC-M53) conjugated to CEN-106, a novel Na,K-ATPase inhibitor known to induce necrosis in human cancer cell types [33].

This study more comprehensively examined the expression of dysadherin in different types of thyroid pathologies and assessed the potential for EDC1 as a treatment for aggressive thyroid cancers by evaluating the efficacy of EDC1 on human thyroid cancer lines.

## RESULTS

### Patients demographics

Characteristics of the 158 subjects represented on the tissue microarray in this study are summarized in Table 1. Among all the cases evaluated, there were 10 normal thyroids, 10 nodular goiters, 11 Hashimoto's thyroiditis (HT), 32 follicular adenomas (FA), 28 papillary carcinomas (PTC), 29 follicular variant papillary carcinoma (FV-PTC), 28 follicular carcinomas (FTC) and 10 anaplastic carcinomas (ATC), with a total of 63 benign and 95 malignant thyroid pathologies. Female predominance was seen in each group. As expected, the ATC patients were older (mean age of 70 years old) than patients with HT, FA, and well-differentiated thyroid carcinomas.

**Table 1:** Patient demographics with respect of pathology

Pathology	No. of Cases	Age (median, range)	Gender (female, %)
Normal thyroid	10	56 (34−83)	7 (70)
Nodular goiter	10	55 (30−67)	6 (60)
HT	11	49 (29−61)	10 (91)
FA	32	48 (16−77)	24 (75)
PTC	28	51 (14−83)	21 (75)
FV-PTC	29	47 (25−76)	20 (69)
FTC	28	59 (18−83)	17 (61)
ATC	10	70 (31−86)	6 (60)

HT, Hashimoto's thyroiditis; FA, follicular adenoma; PTC, papillary thyroid carcinoma; FV-PTC, follicular variant of papillary thyroid carcinoma; FTC, follicular thyroid carcinoma; ATC, anaplastic thyroid carcinoma

### Expression in different pathological types and representative patterns

**Figure 1:**
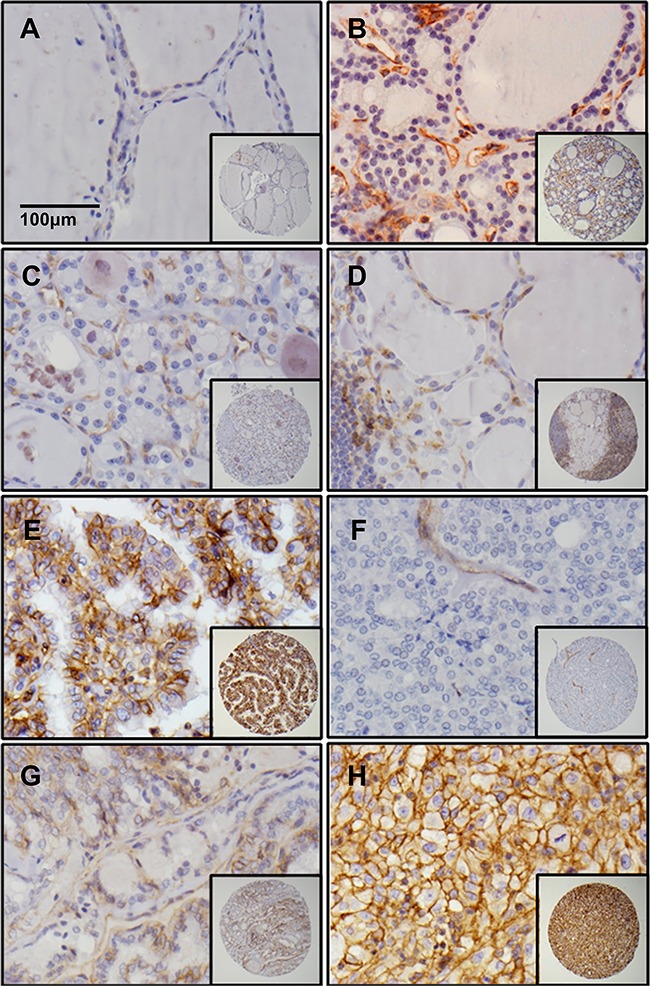
Dysadherin is only expressed in malignant thyroid tissues Immunohistochemistry for dysadherin in core thyroid biopsies of normal thyroid **A**. nodular goiter **B**. follicular adenoma **C**. Hashimoto's thyroiditis **D**. PTC **E**. FTC **F**. FV-PTC **G**. and ATC **H**. The stained samples were scored by two independent pathologists.

The most representative areas of the formalin-fixed paraffin-embedded thyroid tissues were assembled in tissue microarray (TMA) and the expression of dysadherin was examined by immunohistochemistry. Table 2 lists the most common intensity of dysadherin in all the positive cases observed in the different types of thyroid carcinomas. Dysadherin expression was not detectable in most non-malignant thyroid tissues including all normal thyroid, Hashimoto's thyroiditis, and follicular adenoma (representative tissue samples shown in Figure 1A, 1C and 1D, respectively). Only 1 out of 10 nodular goiter showed a weak dysadherin expression (1+) in the cell membrane of thyroid follicular cells (Figure 1B). On the other hand, most of the malignant thyroid tumors (59/95) demonstrated moderate (2+) to strong (3+) cell membrane immunoreactivity for dysadherin (62% vs. 2% in benign group, p<0.001) with diffused pattern localized predominantly to the cell membrane. Dysadherin was strongly expressed (3+) in all ATCs (100%, Figure 1H) while moderate positivity (2+) was seen in 7 FTCs (25%, Figure 1F). ATC showed the most intense and the most diffuse pattern of dysadherin expression. A total of 42 PTCs (74%) showed positive staining for dysadherin including 24 classic PTCs and 18 FV-PTCs. A slight expression difference of dysadherin was seen between classical and follicular variant of PTC. Less patients with follicular variant showed dysadherin positivity (86% vs. 62% positivity, p=0.043), and the average of the intensity was weaker (1+) compared with classical PTC (2+) (Figure 1G vs. 1E).

**Table 2:** Dysadherin expression with respect to pathology

Pathology	Positive cases (%)	Mode score
Normal thyroid	0 (0)	(N.A)
Nodular goiter	1 (10)	1+
HT	0 (0)	(N.A)
FA	0 (0)	(N.A)
PTC	24 (86)	2+
FV-PTC	18 (62)	1+
FTC	7 (25)	2+
ATC	10 (100)	3+

HT, Hashimoto's thyroiditis; FA, follicular adenoma; PTC, papillary thyroid carcinoma; FV-PTC, follicular variant of papillary thyroid carcinoma; FTC, follicular thyroid carcinoma; ATC, anaplastic thyroid carcinoma

### Clinical relevance of dysadherin expression

The clinical utility of using dysadherin expression as a potential marker for aggressive characteristics of thyroid cancer and adverse clinical outcomes was examined. Our relatively larger sample size of PTC tissues allowed for the correlation of tumor characteristics with dysadherin expression within the same pathology. Majority of classical and follicular variant of PTC (86% and 62% positivity respectively) overexpressed dysadherin. The clinicopathological correlations with dysadherin overexpression in PTCs are summarized in Table 3. In PTCs, the overexpression of dysadherin was significantly associated with the presence of extrathyroidal extension and lymph node metastases (p=0.045 and p=0.017 respectively), but not with older age, gender, tumor size, focality, or distant metastases. For both extrathyroidal extension and lymph node metastasis, positive expression of dysadherin was seen in 93% and 95% of the cases respectively. However they also exhibited moderate false positive rate (67% and 63% respectively).

**Table 3:** Correlations between clinicopathologic findings in PTC and dysadherin expression

Variables	No.	Positive (%)	p-value
Age, years			
< 45	23	15 (65)	
≥ 45	34	27 (79)	0.232
Sex			
Male	16	11 (69)	
Female	41	31 (76)	0.597
Tumor Size			
<4cm	48	35 (73)	
≥4cm	9	7 (78)	0.761
Multifocality			
Negative	34	24 (71)	
Positive	23	18 (78)	0.519
Extrathyroidal extension			
Negative	42	28 (67)	
Positive	15	14 (93)	*0.045
Lymph node metastases			
Negative	38	24 (63)	
Yes	19	18 (95)	*0.017
Distant metastases			
Negative	56	41 (73)	
Positive	1	1 (100)	0.547

* Statistically significant (p<0.05)

### Dysadherin expressed in human thyroid cancer cell lines

**Figure 2:**
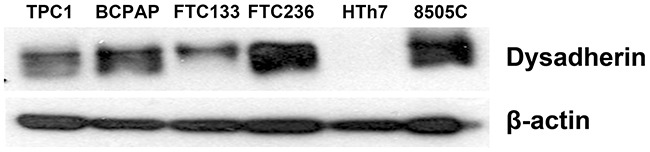
Dysadherin is expressed in human thyroid cancer cell lines Western blot analysis was used to detect dysadherin in six human thyroid cancer cell lines. The majority of thyroid cancers have high protein levels of dysadherin. Beta-actin served as loading control.

Six established unique human thyroid cancer cell lines were tested for the baseline level expression of dysadherin as a potential target for ADCs. Western blot using mAb NCC-M53, a monoclonal antibody against dysadherin, showed that dysadherin is highly expressed in papillary (TPC and BCPAP), follicular (FTC236) and anaplastic (8505C) thyroid cancer cell lines (Figure 2), consistent with the immunohistochemistry data. However, there was a moderate expression of dysadherin in FTC133 and no expression in HTh7 thyroid cancer cells.

**Figure 3:**
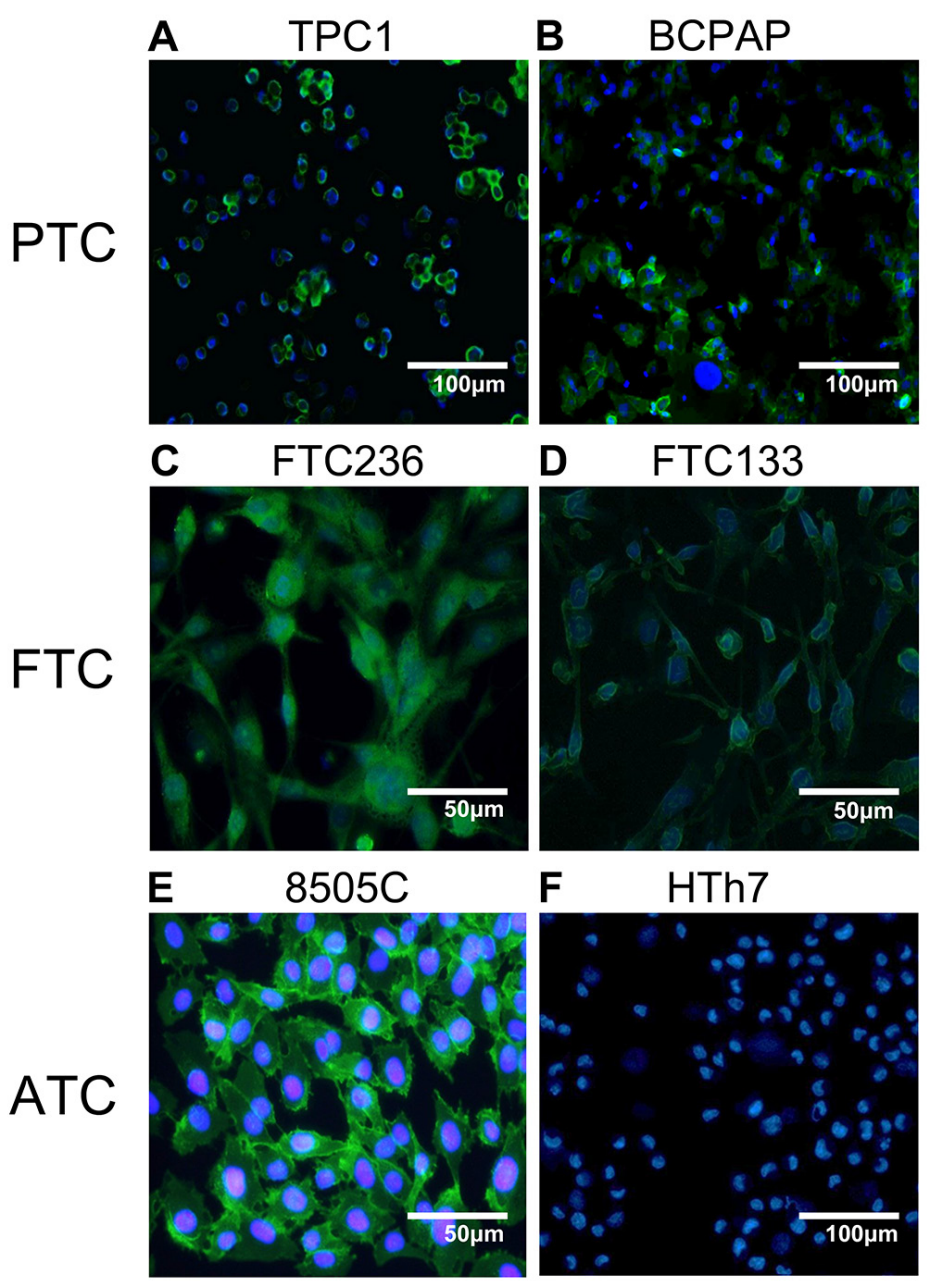
Immunofluoresence of dysadherin in human thyroid cancer cell lines Results from the Western blot analysis was confirmed with the visualization of dysadherin (green) with immunofluorescence. DAPI was used for nucleus counter staining.

We also characterized the human thyroid cancer cells by immunofluorescence for dysadherin. As shown in Figure 3E, 8505c cells have a strong specific immunofluorescence signal, both in the cytoplasm and the cell membrane, while HTh7 cells (Figure 3F) have a markedly diminished immunofluorescence, which is consistent with the Western blot results. Both papillary and follicular cell lines expressed a more modest amount of immunofluorescence. All the cell lines that expressed dysadherin stained positively in the cytoplasm, but TPC1 and 8505c cells exhibited a stronger signal from the cell membrane. As expected, there was no immunofluorescence in the negative controls of the respective cell lines.

### EDC1 inhibited growth in dysadherin positive cell lines

**Figure 4:**
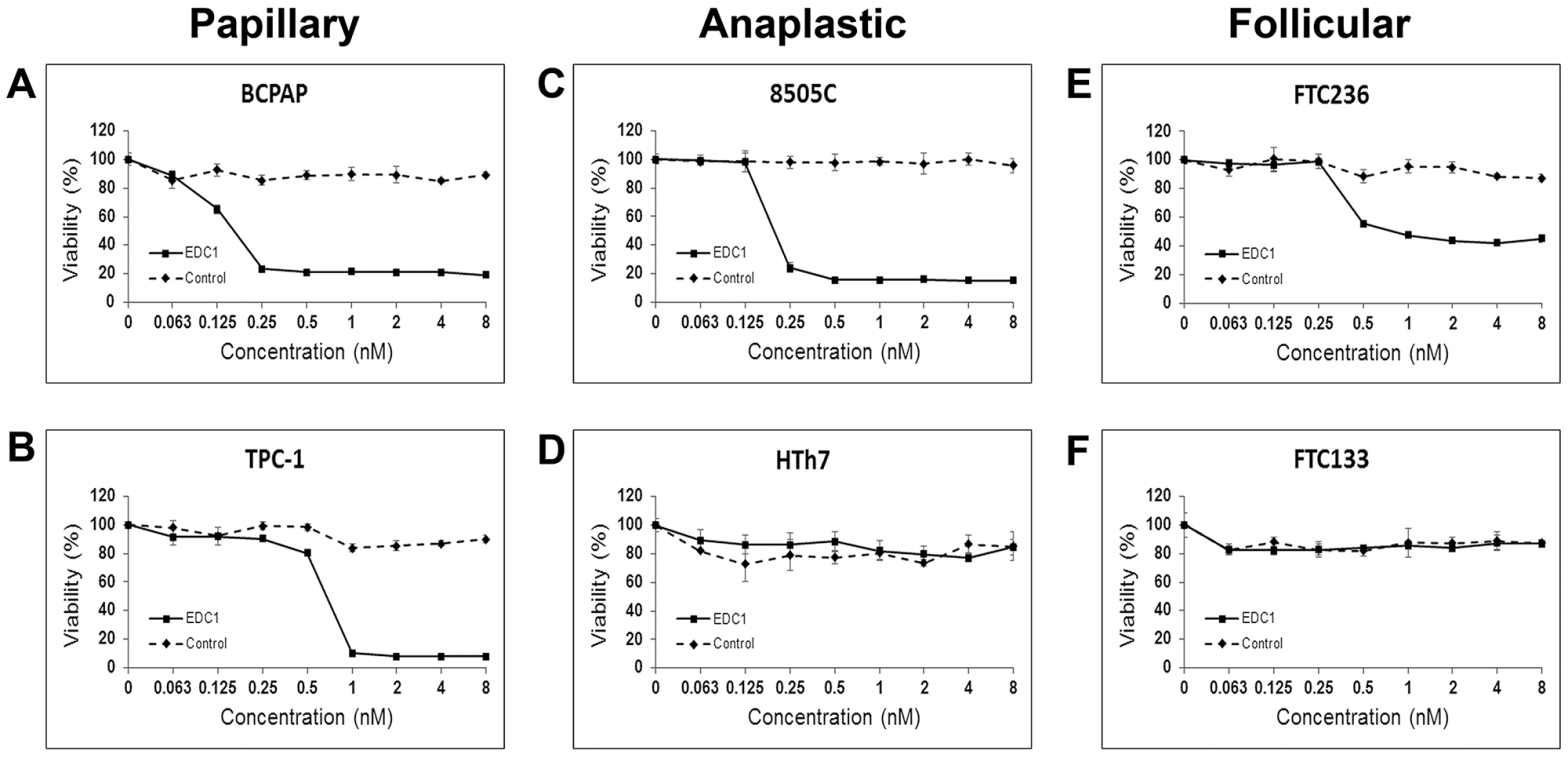
EDC1, an antibody-drug conjugate that target dysadherin, inhibits thyroid cancer cell proliferation The papillary **A** and **B**. anaplastic **C** and **D**. and follicular **E** and **F**. thyroid cancer cell lines were treated with EDC1 (0-8nM) and negative control, the warhead drug conjugated to a non-specific antibody, for 72 hours. Cell viability was measured by a 3-(4,5-dimethylthiazol-2-yl)-2,5-diphenyltetrazolium bromide (MTT) assay. The data was presented in mean percent viability ± SEM.

We hypothesized that while EDC1 would target the warhead drug to dysadherin expression cell, the warhead drug conjugated to a non-specific polyclonal antibody (negative control) would be less cytotoxic to thyroid cancer cells. To demonstrate the potency of EDC1 on thyroid cancer cytotoxicity, we examined the six human thyroid cancer cell lines using the MTT cellular growth assay. All the significantly affected cell lines achieved their half of the maximal inhibitory effect (EC_50_) in the nanomolar range of ECD1 and expressed at least a moderate amount of dysadherin on Western blot analysis (Figure 2). Both 8505c (ATC) and BCPAP (PTC) thyroid cancer cell lines achieved EC_50_ at doses below 0.2 nM (Figure 1A and 1C). As previously noted, these cell lines demonstrated strong expression of dysadherin on their cell membranes. TPC1 (PTC) and FTC236 (FTC) thyroid cancer cell lines showed moderate expression of dysadherin and were sensitive to EDC1, demonstrating EC_50_ below 1.7nM (Figure 4B and 4E). No effect was observed in FTC133 which moderately expressed dysadherin (Figure 4F).

To show that the growth suppressive effect of EDC1 is specific to dysadherin positive cells, an MTT assay was also performed on a dysadherin non-expressing cell line HTh7 (Figure 4D). As expected, EDC1 in doses as high as 8nM had no effect on cellular proliferation in HTh7. The antibody by itself has already been demonstrated to have no effect [33]. In addition, when all thyroid cancer cell lines were treated with the same drug that was conjugated to a non-specific antibody (negative control), no effect was observed on cell proliferation in any of the six thyroid cancer cell lines as expected. This illustrates that the cytotoxicity of the warhead drug is limited by conjugation to an antibody, and that its anti-proliferative effect only occurs in cancer cells that specifically express the target of the conjugated antibody.

**Figure 5:**
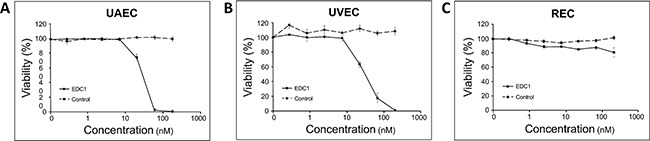
EDC1 has minimal effects on the proliferation of non-cancerous cells Human umbilical artery endothelial **A**. umbilical vein endothelial **B**. and renal epithelial (B) cell lines were treated with EDC1 (0-200nM) for 72 hours. Cell viability was measured by Cell Titer-Glo assay. The data was presented in mean percent viability ± SEM.

When human umbilical artery endothelial cell line (UAEC), umbilical vein endothelial cell line (UVEC), and renal epithelial cell line (REC) were treated with EDC1 in the same manner (Figure 5), very limited cytotoxicity was observed (EC50 = 52nM, 37nM, >100nM respectively) with the EC50 much higher compared to those of the thyroid cancer cell lines. Taken together, these results suggest that EDC1 is more potent against thyroid cancer cells than against non-cancerous cells.

## DISCUSSION

The incidence rate of thyroid cancer more than doubled since 1999 and more than tripled since 1973, reaching 15.07 per 100,000 [34]. An increase has been also evident worldwide [35]. Approximately 90% are classified as well-differentiated, including PTC and FTC [36]. The most common variant of PTC is the follicular variant of papillary thyroid carcinoma (FV-PTC) which may account up to 41% of PTCs [2]. While these well-differentiated thyroid cancers are highly treatable, undifferentiated thyroid cancers such as ATC and 20% of well-differentiated thyroid cancers do not respond to conventional treatments, manifest with metastatic and/or recurrent disease, and lead to increased mortality [3, 6]. Though anaplastic cancers only make up less than 2% of all the cases of thyroid cancer, they represent over half of the thyroid cancer-related deaths, and the mean survival time is less than 6 months from diagnosis [5]. These aggressive thyroid cancers are still a clinical challenge and the research of new treatment strategies is ongoing.

The cell surface molecule dysadherin gained a widespread attention after the discovery that its overexpression is highly associated with metastatic potential and poorer prognosis in breast cancers [37]. Subsequent studies established the expression patterns and clinical correlations of dysadherin in many other cancers, exploring its value as a potential drug target and/or prognostic marker [32]. For example, studies examining squamous cell carcinoma [29] and cutaneous malignant melanoma [27] showed that dysadherin expression is an indepdentent predictor of poor survival. In majority of the cancers, dysadherin expression is correlated with local invasion, distant metastasis, and higher tumor grade [20–30]. In our current study, we show that dysadherin is highly expressed in all ATCs, moderately expressed in tissue samples of PTCs (85%) including FV-PTCs (62%), and seldom expressed in FTC (25%), which are consistent with a previous study [19]. We also demonstrate that these immunohistochemistry findings are consistent with Western blot and immunofluorescence detection of dysadherin in human thyroid cancer cell lines. Furthermore, dysadherin expression in PTCs was associated with extrathyroidal extension and lymph node metastases, but not with older age, tumor size, focality, or distant metastases.

Previous studies have shown that both differentiated and undifferentiated thyroid cancers also express dysadherin [19, 38] and that its expression is higher in aggressive undifferentiated thyroid cancers compared to the differentiated subtypes. In these studies, its expression correlated with tumor size, distant metastasis, regional lymph node metastasis, and disease specific survival in thyroid cancer, which is similar to the findings in our current study with the exception of the negative association of dysadherin overexpression with tumor size and distant metastases. The difference may stem from the fact that the previous study correlated the presence of distance metastasis and tumor size with the degree of dysadherin expression across both differentiated and aggressive undifferentiated thyroid cancers while our study solely analyzed PTCs which are known to be well-differentiated and unaggressive. In our study only one of 57 PTCs showed distant metastases, which overexpressed dysadherin.

Given the association between dysadherin expression and aggressive thyroid cancers and the limited expression of dysadherin in normal cells, dysadherin could potentially serve as an additional adverse prognostic marker for thyroid cancers. However, dysadherin expression may need to be interpreted in conjunction with other factors such as E-cadherin status, another cell membrane protein associated with dysadherin whose dysfunction lead to progression of benign tumor to aggressive metastatic cancer [39]. In another study, E-cadherin level was found to be lower than dysadherin level in undifferentiated thyroid cancers but higher in differentiated thyroid cancers [19]. Dysadherin seem to downregulate E-cadherin, and the measurement of E-cadherin expression can improve the prognostic power of dysadherin expression [32]. Regarding the value of using dysadherin expression as a prognostic marker, differentiated and undifferentiated thyroid cancers perhaps are best analyzed separately because of their different behaviors, pathologies, and relative E-cadherin expression which may affect metastatic potential and prognostic utility [28, 38].

Dysadherin expression has another clinical utility in that it is expressed predominantly on cancer cells and almost never in normal cells, which makes it an attractive target for targeted drug therapy including EDCs [33]. EDCs offer a theoretical synergy of mechanically combining the tumor-targeting properties of antibodies to the potent cytotoxic effect of a linked molecule leading to increased therapeutic potential while avoiding its harmful effects on normal cells. In the present study we show that no normal thyroid tissue express dysadherin and only 1 of 53 benign thyroid conditions (nodular goiter, Hashimoto's thyroiditis, and follicular adenoma) weakly expressed dysadherin while majority of thyroid malignancies showed moderate or strong expression. We also show that dysadherin is overexpressed in the majority of aggressive thyroid cell lines.

Using the overexpressed dysadherin on aggressive thyroid cancers as a target, a new anti-thyroid cancer therapy was created using the EDC technology. EDC1 is a novel conjugate composed of a novel inhibitor of the Na,K-ATPase (CEN-106) conjugated to an anti-dysadherin antibody via a long non-cleavable linker. While other Na,K-ATPase inhibitors such as digitoxin have been used for the treatment of heart pathologies for centuries, it was not until 1964 when researchers discovered that a class of naturally occuring inhibitors of the Na,K-ATPase known as cardiac glycosides were potent anti-cancer drugs [40, 41]. This line of research eventually lead to a series of Phase I clinical trials examining such Na,K-ATPase inhibitors for the treatment of cancer. Unfortunately, these trials all failed to show significant clinical benefit, most likely due to the fact that all cells express the Na,K-ATPase. Therefore, to better target the Na,K-ATPase inhibitory power of CEN-106, we conjugated it to a targeting antibody. Our choice of the EDC1 antibody stem from the discovery that dysadherin is a subunit of the Na,K-ATPase [42, 43] and overexpressed on metastatic cancer cells [37]. We have shown previously that CEN-106 is active on numerous human cells with EC50s between 1 and 5nM [33]. Conjugates where the antibody bound to targets expressed on cells but not co-localized with the Na,K-ATPase were shown to have very poor activity *in vitro* and *in vivo* on multiple cell lines. Together, these two extracellular targets make EDC1 both highly potent and highly specific.

Our cell proliferation assays demonstrate that in sub-nanomolar concentrations, EDC1 shows selective and dose dependent inhibition of thyroid cancer cell growth when they express moderate to high levels of dysadherin, leaving cells that do not express dysadherin unharmed. Fortunately, EDC1 showed a very limited cytotoxic effect against non-cancerous cells. The targeted nature of EDCs may favor the use of drugs like EDC1 over more traditional cytotoxic therapies for aggressive thyroid cancers. Interestingly FTC133 which has a moderate expression of dysadherin did not respond to ECD1. A possible explanation is that the level of both the antibody target and the drug target affect the potency of the antibody-drug conjugate. ECD1 is more potent in cells where the expression of Na,K-ATPase is much lower relative to dysadherin; the ratio of the two targets needs to favor the antibody target [33]. We suspect that the dysadherin expression is far less than that of Na,K-ATPase in FTC133.

Despite the above findings, this study does possess some limitation. It is unknown whether the FV-PTC samples in our study are truly FV-PTC or noninvasive follicular thyroid neoplasm with papillary-like nuclear features (NIFTP) based on the new criteria published on August of 2016 [44]. As the tissue microarrays were constructed retrospectively, we were unable to assess all the criteria to classify some FV-PTC as NIFTP. In some cases, the entire capsule was not submitted for microscopic examination which precludes the assessment of capsular invasion. Additionally, in several cases the tumors were encapsulated, but there were multiple tumors present in the same lobe including papillary thyroid microcarcinomas. While the percentage of dysadherin positive FV-PTC and PTC were similar in our study, future studies should classify FV-PTC according to the new criteria.

While we have demonstrated that EDC1 is cytotoxic to thyroid cancer cells in subnanomolar concentrations, another limitation is the lack of data on EDC1′s toxicity *in vivo* since many cardiac glycosides failed clinical trials due to side effects as mentioned earlier. We have previously demonstrated limited drug toxicity in small animals [33]. However, given that mice used in human xenograft studies are immunocompromised and that CEN-106 kill cells by a process resembling necrosis that induce the immune system, we are working on advanced *in vivo* models to better understand the true efficacious activity of these EDCs. Additionally, we are in the processes to test drug toxicities in primates.

In summary, dysadherin is expressed in aggressive thyroid cancers, especially anaplastic thyroid cancers, and its expression is associated with poor prognosis. Additionally, conjugation of anti-dysadherin monoclonal antibodies to novel and potent cytotoxic Na,K-ATPase inhibitors presents an innovative targeting modality for highly effective and safe anticancer agents. Therefore, EDC1 warrants further investigation for treating patients with aggressive thyroid cancers.

## MATERIALS AND METHODS

### Patients

The study was approved by the Institutional Review Board. Patients diagnosed with follicular thyroid carcinoma (FTC), papillary thyroid carcinoma – both the classical (PTC) and the follicular variant (FV-PTC) – and anaplastic thyroid carcinoma (ATC) were identified using a surgical pathology database in UW Hospital, Madison, WI, USA. The hematoxylin and eosin-stained histological slides were reviewed to confirm the initial diagnosis by the attending pathologists at the time of the surgery. The clinical pathologic data from the patients with thyroid cancer were obtained, which included the age at diagnosis, gender, primary surgery type, evidence of metastasis, evidence of recurrence, and the date of last follow-up or death. The information on tumor size, multifocality, extrathyroidal invasion, lymphovascular invasion, and lymph node metastasis was obtained from the surgical pathology reports. The tumors were classified according guidelines published by the American Thyroid Association [45].

All patients received post-operative evaluation using a combination of radioactive iodine, thyroglobulin levels, ultrasound, computed tomography, and fine needle aspiration to detect persistence, distant metastasis, and local recurrence. Persistence was defined as any indications of the primary tumor within six months of the initial surgery. Follow-up duration was calculated up to the time of death, time of last evaluation, or lost to follow-up. Survival data was last collected in June 6, 2014.

### Tissue microarray construction

The most representative areas of the formalin-fixed paraffin-embedded thyroid tumors were organized on a tissue microarray in triplicate 0.6mm cores using manual tissue microarrayer (Beecher Instruments, Sun Prarie, WI, USA) after the confirmation of the thyroid cancer diagnosis. Normal thyroid controls were obtained from tissues incidentally removed from patients undergoing surgery for hyperparathyroidism or from the histologically normal thyroid tissues on the opposite lobe of the malignancy. Thyroid tissues involved in follicular adenoma, nodular goiter, and Hashimoto's thyroiditis were collected for benign thyroid disease control. Sections of 5μm were cut for immunohistochemical analysis. Hematoxylin and eosin stain was used to confirm the placement of the tissue cores on the recipient block of the microarray.

### Immunohistochemistry

Automated immunostaining was done by first deparaffinizing the previously mentioned 5μm thick tissue microarray sections followed by heat-induced epitope retrieval using Lab Vision PT module (Thermo Scientific) with Lab Vision citrate buffer pH 6.0. Immunolabeling was done at room temperature using the Lab Vision 360 LV-1 Autostainer system (Thermo Scientific). All reagents were obtained from Biocare Medical, Chicago IL, USA. Nonspecific protein binding was blocked by Sniper and nonspecific avidin was blocked using the Avidin-Biotin kit, incubating 15min for each reagent. NCC-M53 (antibody to dysadherin) was kindly provided by Dr. Setsuo Hirohashi (National Cancer Center in Tokyo, Japan). The antibodies were incubated for 60 min followed by incubation with biotinylated goat anti-mouse IgG for 15 min and subsequent 4plus Streptavidin–HRP treatment for 15 min. BetazoidDiaminobenzidene and Mayer's Hematoxylin were each incubated for 1 min. Primary antibodies were omitted in negative controls, which resulted in no staining. For positive controls, thyroid tumors previously shown to express the antigen of interest by immunohistochemistry and by RT-PCR.

For manual immunostaining, full tissue sections of the ATC were deparaffinized followed by microwave heat-induced epitope retrieval with 10mM citrate buffer pH 6 for 20 min. Endogenous peroxidase was blocked with 3% hydrogen peroxide for 15 min followed by a nonspecific protein block with a 2.5% normal horse serum for 30 min. The primary antibodies to dysadherin was incubated overnight at 4°C at half the dilution used for automated immunostaining.

The following day the slides were washed and incubated for 30 min with the ImmPRESS Universal Peroxidase Reagent Kit (Vector Laboratories, Burlingame, CA) and developed with the ImmPACT DAB Kit (Vector Laboratories) and Gills Hematoxylin (Vector Laboratories) incubating each for 1 min.

Two independent observers (D.B. and R.V.L.) scored the immunohistochemistry staining of the thyroid cancer tissues using conventional bright field microscopy. Differences in interpretation were reviewed for consensus. The membranous staining for dysadherin was interpreted based on the intensity as negative, weak (1+), moderate (2+) and strong (3+). The pattern of expression was considered restricted, focal, or diffuse if the staining involved less than 2% of all the tumor cells in the tissue microarray sample, 2-25%, and more than 25% respectively.

### Cells and reagents

Human derived thyroid cancer cells TPC-1 (papillary), BCPAP (papillary), FTC133 (follicular), FTC236 (follicular), HTh7 (anaplastic), and 8505C (anaplastic) were used. All six cell lines were authenticated and confirmed with their unique identify by DNA profiling [46]. TPC-1 (provided by Dr. Daniel Ruan, Brigham and Women's Hospital, Boston, MA), HTh7 (provided by Dr. Rebecca Schweppe, University of Colorado, Denver, CO) and 8505C (Dr. Daniel Ruan) were maintained in RPMI 1640 (Invitrogen Life Technologies, Carlsbad, CA) with 10% fetal bovine serum (FBS) (Sigma-Aldrich, St. Louis, MO), and 1% penicillin/streptomycin. FTC133 and FTC236 were purchased from European Collection of Cell Cultures (ECACC) and maintained in DMEM/Ham's F-12 medium (1:1; Invitrogen) with 10% FBS, 5mg/500mL insulin (Sigma-Aldrich), 5u/500ml thyrotrophic hormone from bovine, and 1% penicillin/streptomycin. UAEC, UVEC, and REC cell lines were grown in Endothelial Cell Growth Medium 2 and Renal Epithelial Growth Medium 2 (PromoCell GmbH). All cell lines were grown in 5% CO2 at 37°C.

### Immunofluorescence

The six cell lines previously mentioned were plated on 8-well chamber slides (Nalge Nunc) in RPMI medium containing 10% FBS. The cells were then rinsed and fixed in 4% paraformaldehyde for 30 minutes at room temperature. After permeabilization in 0.2% Triton X-100 for 10 minutes at 4°C and blocking with 5% bovine serum albumin and 3% donkey serum in PBS for 1 hour at room temperature, the cells were incubated with dysadherinmAb NCC-M53 antibodies, diluted 1:1000, overnight at 4°C. Antigens were visualized using Alexa-488-conjugated donkey anti-mouse and Alexa-555–conjugated donkey anti-rabbit (1:300, 30 minutes) secondary antibodies (Invitrogen). Nuclear staining was conducted with DAPI (Invitrogen). Images were acquired with a Nikon Eclipse E800 upright microscope and attached RetigaEXi CCD digital camera. Images were processed and analyzed using Adobe Photoshop 7.0.

### Western blot

Total cellular proteins were isolated from all six human thyroid cancer cell lines as previously described [47]. Protein concentrations were quantified by BCA Protein Assay Kit (Thermo Scientific, Waltham, MA). Denatured proteins (20μg) from each sample were resolved by gel electrophoresis (Bio-Rad Laboratories) and transferred to nitrocellulose membranes (Bio-Rad Laboratories). Protein-bound membranes were blocked in 5% nonfat milk solution and incubated with dysadherinmAb NCC-M53 antibodies, diluted 1:1000, overnight at 4°C. The next day, the nitrocellulose membranes were washed and incubated for 1 hour at room temperature with horseradish peroxidase-conjugated secondary antibodies (Cell Signaling Technology, Beverly, MA). Immunoreactive protein bands were detected by Immunstar (Bio-Rad Laboratories).

### Cell proliferation assays and EC50 calculations

All human thyroid cancer cell lines were treated with EDC1 (produced as described [33]) and/or negative control (CEN-106 conjugated to a non-specific antibody) in varying doses to examine its effect on cell proliferation and to determine the half-maximal inhibitory concentration (EC_50_) with 3-(4,5-dimethylthiazol-2-yl)-2,5-diphenyltetrazolium bromide (MTT) assay. The cell lines were plated in quadruplicates on 24-well plates and incubated overnight to allow cell attachment. EDC1 was added in fresh medium (0-8nM) and incubated for 72 hrs. On the day of measurement, the treatment medium was removed, phenol red-free medium containing 0.5 mg/mL MTT was added to each well and incubated for 3 ½ hours at 37°C. The MTT formazan crystals were dissolved by adding DMSO into each well and the absorbance was measured at 540nm in a spectrophotometer (μQuant; Bio-Tek Instruments, Winooski, VT). The Cell Titer-Glo assays (Promega) were done in non-cancer cell lines in a similar manner. The Cell Titer-Glo Luminescent Cell Viability Assay was performed 72 hrs after treatment. Wallac Victor2 Model 1420-041 assay plate reader (Perkin Elmer, Gaithersburg, MD) was used for luminescence measurements. The EC_50_ values were calculated using GraphPad Prism 6 software (GraphPad).

### Statistics

The data obtained from patient medical records was analyzed using the Statistical Package for Social Sciences (SPSS for Windows, version 17.0; Chicago, IL). Unless specifically noted, continuous data were presented as mean ± SEM. Student t-test (two-sided) and One-way ANOVA were used to evaluate differences between two groups and among multiple groups, respectively. Tests of survival equality were performed by the log-rank test. P <0.05 was considered statistically significant.
